# Metabolism and Chemical Degradation of New Antidiabetic Drugs: A Review of Analytical Approaches for Analysis of Glutides and Gliflozins

**DOI:** 10.3390/biomedicines11082127

**Published:** 2023-07-27

**Authors:** Anna Gumieniczek, Anna Berecka-Rycerz

**Affiliations:** Department of Medicinal Chemistry, Faculty of Pharmacy, Medical University of Lublin, Jaczewskiego 4, 20-090 Lublin, Poland; anna.berecka-rycerz@umlub.pl

**Keywords:** drug metabolism and drug degradation, chromatographic and radiometric methods, in vitro metabolic studies, peptide drugs, glutides and gliptins

## Abstract

The drug metabolism and drug degradation pathways may overlap, resulting in the formation of similar constituents. Therefore, the metabolism data can be helpful for deriving safe levels of degradation impurities and improving the quality of respective pharmaceutical products. The present article contains considerations on possible links between metabolic and degradation pathways for new antidiabetic drugs such as glutides, gliflozins, and gliptins. Special attention was paid to their reported metabolites and identified degradation products. At the same time, many interesting analytical approaches to conducting metabolism as well as degradation experiments were mentioned, including chromatographic methods and radioactive labeling of the drugs. The review addresses the analytical approaches elaborated for examining the metabolism and degradation pathways of glutides, i.e., glucagon like peptide 1 (GLP-1) receptor agonists, and gliflozins, i.e., sodium glucose co-transporter 2 (SGLT2) inhibitors. The problems associated with the chromatographic analysis of the peptide compounds (glutides) and the polar drugs (gliflozins) were addressed. Furthermore, issues related to in vitro experiments and the use of stable isotopes were discussed.

## 1. Introduction

Type 2 diabetes mellitus (T2DM) is one of the most common metabolic diseases. It results from decreased secretion by pancreatic b-cells and insulin resistance. In turn, alterations in insulin acting in peripheral tissues lead to elevated blood glucose levels with severe metabolic disturbances, e.g., macro- and micro-vascular complications. Thus, the treatment of hyperglycemia should target not only impaired insulin secretion but also hepatic and muscular insulin resistance, reduced intestinal incretin effects, and increased glucose renal threshold. Optimal management of T2DM should also consider other risk factors beyond glycemic control, i.e., excess body weight, cardiovascular risk, heart failure, and renal diseases [1,2,3]. Therefore, typical initial drugs to treat T2DM, i.e., insulin and sulfonylureas, are not optimal for every patient because of the associated risk of hypoglycemia and weight gain. An increase in body weight in diabetic patients potentially leads to increased insulin resistance and cardiovascular risk. Therefore, the preferred drugs are those that do not increase or even lower the body weight, such as glucagon-like peptide 1 (GLP-1) receptor agonists (GLP-1 RAs or glutides) and sodium glucose co-transporter 2 inhibitors (SGLT2 inhibitors or gliflozins). Therefore, combined treatment with a GLP-1 RA and an SGLT2 inhibitor, with or without metformin, could be the recommended initial therapy for T2DM for many patients. As a result, in addition to lowering glucose levels, a decrease in cardiac events, cardiac mortality, and the rate of kidney damage could be expected [1,4,5].

Drug metabolism is a complicated process in which drugs can be modified by various endogenous enzymes. Experiments on the drug metabolism are important steps in the process of modifying new molecules for their optimal therapeutic properties, identifying new active compounds based on the structures of active metabolites, enhancing drug safety due to the elimination of toxic metabolites, and comparing the drug metabolism in animals and humans for predicting optimal human doses [6,7,8]. Despite the human and animal studies, in vitro metabolism experiments, which are relatively inexpensive and easy to carry out, could be used as a reliable screening procedure to recognize the drug metabolites, which could explain the particular metabolic pathways and designate directions for further in vivo experiments. These in vitro models include the use of slices from liver and kidney tissues, recombinant human cytochromes (CYPs), other enzymes, chemical inhibitors, and co-factors. Essential points for conducting such in vitro studies include examination of appropriate concentrations of substrates, co-factors, enzymes and their inhibitors, and, finally, the drugs being tested. In addition, the important steps of experiments, such as the time of incubation and time of sampling, as well as all steps of method validation, should be determined with great care. Finally, the data from in vitro experiments should be analyzed in relation to animal and human studies to predict the consequences of drug metabolism in patients. After an incubation with enzymes and all needed ingredients, the samples from these in vitro experiments are usually analyzed with high-performance liquid chromatography combined with high-resolution mass spectrometry (HPLC-HRMS). This combination is used because of its excellent sensitivity, selectivity, accuracy, and precision of quantification. The accurate mass measurement allows identification of individual components without the need to separate them, simplifying analysis and interpretation of the results [9]. The next step of such experiments is the investigation of the structures of the detected metabolites in relation to their parent drug, in which the main metabolic pathways of phase I, i.e., oxidation, reduction, and hydrolysis, and phase II, i.e., acetylation, sulfation, and glucuronidation, should be taken into account. Finally, in vitro metabolism studies in human and animal tissues (e.g., liver or kidney slices) together with in vivo metabolism studies in animals are useful approaches for identifying major metabolism pathways, i.e., “soft spots” of the examined drugs [7,8].

Even with the possibility of using various software and databases, the identification of unknown compounds, including metabolites or degradation products, is still a complex task. The measurement of accurate masses, the correlation of retention values from chromatography, and fragmentation spectra from MS are often difficult and may be insufficient for the complete identification of many compounds. For reliable identification of metabolites, it is usually necessary to increase the concentration of the analytes and carry out counter-synthesis. Thus, concurrent experiments with or without inserting particular isotope labels should be carried out, as it is a credible tool for the identification of these unknown molecules. The stable isotope labeling provides a huge elevation in both metabolite identification and metabolic pathway proposal. Such stable isotopes have the same number of protons as common elements, and consequently share the same physicochemical properties, but they differ in mass due to a difference in the number of neutrons. Among biochemically relevant elements, carbon, hydrogen, nitrogen, oxygen, and sulfur that have two or more stable isotopes with measurable abundance can be used in the labeling of the samples. For example, carbon (C) is found predominantly as the light isotope 12C (98.89% abundance), but also in the form of a heavy stable isotope 13C (1.11%), with an additional neutron, in addition to trace amounts of a radioactive heavy isotope 14C [10]. The heavier isotopes within the labeled substrates are converted into metabolites, and mixtures of isotopes are created. Metabolites that contain stable isotopes and their respective unlabeled counterparts have the same chemical formulas and structures. Generally, they behave identically during chromatographic separation. However, such compounds can be readily differentiated by their *m*/*z* when a mass spectrometer is used. Finally, the relative amounts of labeled and unlabeled compounds, as well as the extent of labeling within metabolites, can be used to determine the rate of metabolic reactions [7,8,9]. The major advantages of incorporating stable isotope labeling in such experiments is being able to understand the known pathways as well as identify the novel ones. Labeling with stable isotopes is one of the most commonly used procedures in the study of the metabolism of many new drugs, including the drugs studied in the present review, i.e., GLP-1 RAs and SGLT2 inhibitors.

Chromatographic techniques still make up the majority of the methods used for the separation, isolation, and purification of drugs and their metabolites from biological samples, as well as their degradation products. However, after the separation and isolation are accomplished, specialized procedures and techniques are required for the identification of the particular products. The standard spectroscopic techniques, such as ultraviolet absorption (UV), mass spectrometry (MS), infrared spectroscopy (IR), and nuclear magnetic resonance (NMR), may be used off-line after metabolite or degradant isolation or coupled with liquid (HPLC or LC) or gas (GC) chromatographic methods. Today, the GC-MS and LC-MS methods are the prevailing analytic tools in metabolism and degradation studies. Compared with GC-MS, the advantage of LC-MS is that there is no need for chemical derivatization and, generally, faster run times for the analytes. With the introduction of ultra-performance liquid chromatography (UPLC) and highly accurate MS, the application of UPLC-MS has tremendously increased when compared with traditional methods [6,11].

Our present review is focused on some analytical procedures in the LC, UPLC, and MS methods that have impacted the identification of metabolites and degradants of the drugs from two important groups of antidiabetic drugs, i.e., GLP-1 RAs (glutides), which are the peptide compounds, and SGLT2 inhibitors (gliflozins), with highly polar groups from sugar moieties [6,12,13]. The analysis of these antidiabetics and their metabolites or degradation products is challenging, especially with multi-targeted methods. Many polar analytes, e.g., peptides, as well as amino acids and sugars derived from metabolism or degradation, might be co-isolated during the sample preparation and co-eluted, interfering with separation and/or detection processes [14]. All the methods from the cited papers, i.e., column and thin layer liquid chromatography (LC and TLC) and liquid chromatography coupled with mass spectrometry (LC-MS), were briefly mentioned in the text, while the analytical details for respective LC-MS, LC-MS/MS, or high resolution mass spectrometry (HRMS) methods are additionally described in respective tables. There is no similar review in the literature concerning these beneficial antidiabetic drugs in terms of their metabolism and chemical degradation as well. That is why we drew attention to these important issues, and at the same time, we discussed the most interesting analytical procedures that can be used for such experiments. Thus, the present review could be valuable for researchers working on drug metabolism or stability and on the biological impact of particular metabolites or degradants.

## 2. Analytical Tools for Peptide Drugs Examination

The best methods for the separation of peptide products include HPLC, UPLC, ion exchange high performance liquid chromatography (HPLC-IEX), and size exclusion high-performance liquid chromatography (HPLC-SEC). Furthermore, the high sensitivity and selectivity of MS coupled with LC or UPLC (HRMS) are suitable for the identification of peptides, their metabolites, and degradation products. For peptide compounds, the most widely used ionization modes in MS are electrospray ionization (ESI) and matrix-assisted laser desorption ionization (MALDI) techniques [15]. Mass spectrometers can also be combined with a quadrupole, such as a Q-TOF system, in which fragmentation can increase the selectivity of these methods. Furthermore, ion trap and orbitrap mass analyzers that deliver excellent resolution and mass accuracy could be applied. These accurate HRMS systems detect a wide range of compounds as well as small molecules during both targeted and untargeted analyses without losing selectivity or sensitivity. Due to its high specificity, HRMS allows measuring the majority of peptides, including those with changed sequences, even if they are not completely separated. Even when changes to the primary amino acid sequence in peptides due to degradation or other changes occur, they could be recorded as detectable mass shifts. Furthermore, metabolomics studies of peptide drugs are frequently performed using HRMS [11,15]. 

Generally, peptides can be separated with the most frequently used C18 columns; however, some of them can give poor peak shape or peak tailing. For these reasons, C4 columns are recommended for the separation of peptides. Furthermore, phenyl columns, which are similar to C4 columns as far as their hydrophobicity is concerned, could be used for peptide analysis. In the literature, the usefulness of polar embedded or polar endcapped columns, in which polar interactions between peptides and the particle surface are enhanced, is also reported. In addition, peptide drugs could be more effectively separated using longer columns than those usually used in the separation of small drug molecules. Thus, columns with a length of 15 or 25 cm are recommended. As far as mobile phases are concerned, the reversed phase (RP) mode of chromatography could be used for peptides, but it usually requires the use of ion-pair reagents in order to achieve a good peak shape for the analytes [16]. Among the best-rated ion pair reagents for such analysis is trifluoroacetic acid (TFA), which can be added to the mobile phase at a concentration of 0.1%. In consequence, the mobile phases used for peptide separations in RP systems and containing TFA are generally adjusted to a low pH. At such conditions, the carboxylic groups of amine acids are non-ionized and only slightly polar. On the other hand, when the pH of the mobile phase rises to 6–7, the carboxylic groups tend to ionize, making the peptide less hydrophobic. This ionization process reduces the retention of all peptides, but particularly affects those that contain aspartic or glutamic acids, thus changing selectivity in such separations [13,16].

To carry out a forced degradation study of macromolecules, generally, temperature, pH change, and agitation are used as factors to check the instability of the desired molecules. However, small peptides should be subjected to acidic, basic, and neutral hydrolysis, oxidation, photostability, and thermal treatment, according to the strategy of ICH guidelines Q1A(R2) [17], just like the small molecule drugs. At the same time, the selection of appropriate stress conditions is essential and constitutes the most crucial step in conducting the decisive forced degradation study. It is recommended to optimize the stress conditions in such a way to attain degradation kinetics thermodynamically equivalent to accelerated stability conditions [18]. It is known that excessive stress can lead to the secondary degradation of products. On the other hand, inadequate stress will not resolve the purpose of the forced degradation study. For peptide drug substances, the degradation rate and extent depend on the nature and position of amino acids present in their sequences. Therefore, stress conditions can vary on a case-by-case basis. There is neither a practical protocol nor any specification limits defined for the worthwhile forced degradation study of peptides. However, it is accepted that stress conditions for peptide drugs should be set to achieve 5–20% degradation, identically to those for small molecule drugs [19].

## 3. Glutides (GLP-1 RAs)

To ameliorate the altered incretin effects in T2DM, glutides or the GLP-1 RAs, i.e., albiglutide (ALBI), dulaglutide (DULA), exenatide (EXE), liraglutide (LIRA), lixisenatide (LIXI) in injectable formulations, and semaglutide (SEMA) in injectable and oral formulations, are introduced as effective therapeutic approaches. It is well known that the incretin effect contributes nearly half of the insulin secretory response to oral glucose load and that this effect is reduced along with worsening glucose tolerance in diabetic patients. The glucose-dependent insulinotropic polypeptide, formerly known as gastric inhibitory peptide (GIP), and glucagon-like peptide-1 (GLP-1) are the two primary incretins secreted from the intestine on ingestion of glucose to stimulate insulin secretion from pancreatic b-cells, enhance satiety, and delay gastric emptying. GIP and GLP-1 exert their effects by binding to their specific receptors, the GIP receptor and the GLP-1 receptor (GLP-1 R), which both belong to the G-protein-coupled receptor family. Physiologically, GIP and GLP-1 are rapidly degraded by the enzyme dipeptidyl peptidase 4 (DPP-4), which acts on peptides to cleave the two NH_2_-terminal amino acids [20,21]. Thus, GIP and GLP-1 share common properties as incretins, but they also possess different biological characteristics. Endogenous GIP exerts strong insulinotropic effects in healthy subjects, but its insulinotropic effect is seriously reduced in diabetic patients, which discourages the development of GIP-based therapies. In contrast, the insulinotropic effect of GLP-1 is preserved in T2DM patients, establishing GLP-1 and GLP-1R signaling as attractive therapeutic targets [21]. GLP-1 RAs exert a dual action on the endocrine pancreas cells, with a stimulation of insulin secretion by b-cells, mainly in the postprandial state, and an inhibition of glucagon secretion by a-cells, contributing to reducing hyperglycemia. In addition, they slow down gastric emptying and activate the hindbrain GPL-1 receptors. The above effects of GLP-1 RAs can increase satiety and reduce food intake, thus contributing to weight loss in obese diabetic patients. As their effects are glucose-dependent, the risk of hypoglycemia is limited, which is one of their most important properties [20,21]. 

All glutides present on the market are synthetic peptides with high homology with human GLP-1 (7–37). EXE, which is given to patients subcutaneously twice daily, was the first GLP-1 RA introduced into therapy in 2005 (FDA approval). It is a 39-amino acid synthetic peptide with 53% homology with human GLP-1 (7–37). It contains an Ala8Gly substitution that increases its resistance to degradation by DPP-4. In addition, EXE in the form of a sustained-release formulation that could be given subcutaneously once a week is also available on the market. It consists of an EXE moiety embedded within biodegradable polymeric microspheres of poly(DL-lactic-co-glycolic acid) [22]. 

Next GLP-1 RAs, including subcutaneously once daily administered LIRA and LIXI and once weekly given ALBI and DULA, were approved by FDA in 2021, 2016, and 2014, respectively. LIRA is a true analogue of GLP-1 (97% homology) with the addition of a 16-carbon palmitic acid chain conjugated via a glutamate spacer at Lys in position 26, to mask the DPP-4 cleavage site. LIXI is an exendin-4 analogue (94% sequence homology with GLP-1) with 6 Lys residues added to allow resistance to DPP-4. ALBI consists of 2 copies of GLP-1, each with an Ala8Gly substitution, and as a whole, the molecule is fused to albumin [23]. DULA has two copies of a GLP-1 analogue (with amino acid substitutions Ala8Gly, Gly22Glu, and Arg36Gly) that are covalently linked to an Fc fragment of human IgG4 [24]. 

**Figure 1:**
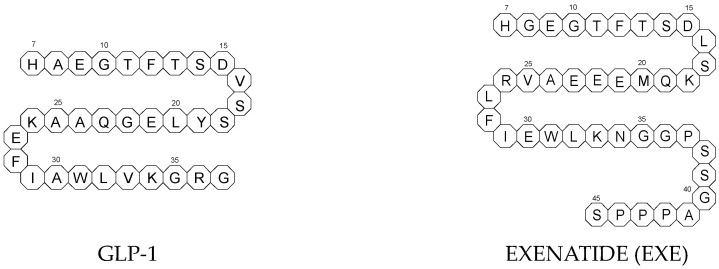

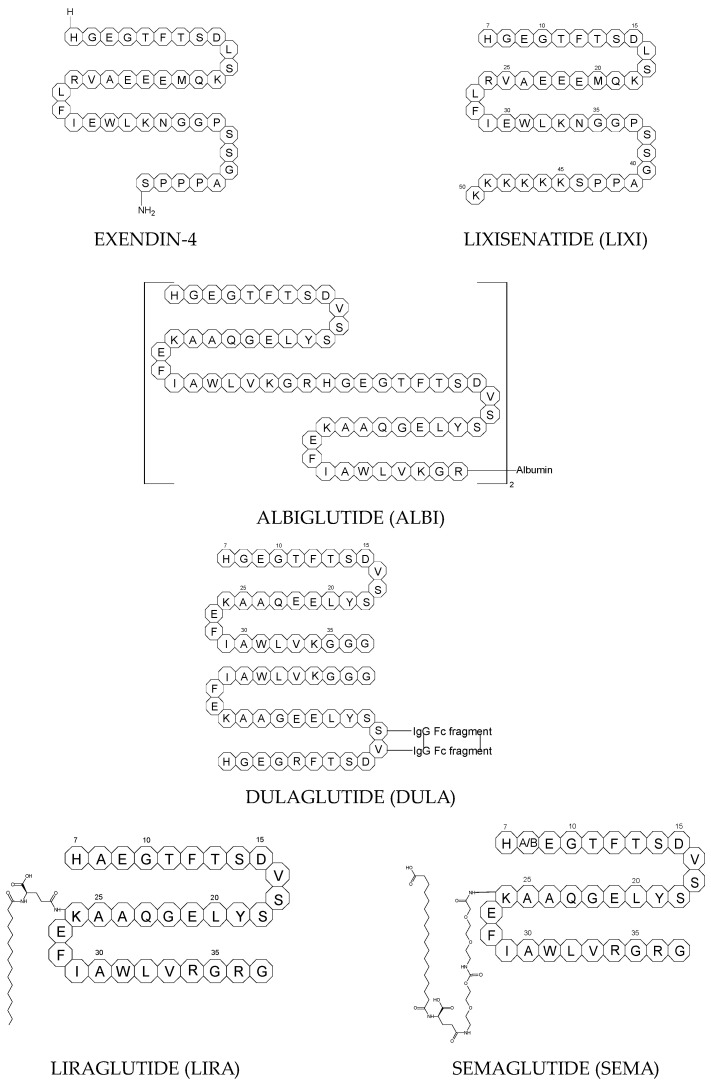
Chemical structures of GLP-1 RAs agonists: albiglutide (ALBI), dulaglutide (DULA), GLP-1, exenatide (EXE), exendin-4, liraglutide (LIRA), lixisenatide (LIXI) and semaglutide (SEMA).

The next GLP-1 RA, i.e., SEMA, is marketed as a once-weekly subcutaneous injection and was approved by the FDA in 2017. It is an analogue of LIRA with a substitution of Ala at position 8 with an aminoisobutyric acid (A/b). The C16 fatty acid is also exchanged for the C18 fatty acid and linked by a synthetic spacer. Acylation with a spacer and C-18 fatty acid chain increases its binding to blood albumin, which enables its longer presence in the blood circulation. Despite the above-mentioned subcutaneous form of SEMA, its orally administered analog with the absorption enhancer sodium N-(8-[2-hydroxybenzoyl]amino)caprylate (SNAC) to overcome the problems of poor absorption and degradation in the stomach was approved in 2019 [21,25]. The structures of the mentioned GLP-1 RAs are presented in Figure 1.

### 3.1. Metabolic Transformations of Glutides (GLP-1 RAs) and the Methods Used to Examine Their Metabolic Pathways

The HRMS method was used to assess the metabolism of different GLP-1 RAs, i.e., exenatide, lixisenatide, liraglutide, and semaglutide (EXE, LIXI, LIRA, and SEMA) in subcutaneous tissue of rats, minipigs, and humans [26]. For the separation of these drugs and their metabolites, two types of columns were used, depending on the analyzed peptide, i.e., XSelect CSH C18 XP and UPLC Peptide CSH C18 (Waters, Milford, MA, USA). Analyses were conducted with an orbitrap mass spectrometer operating in ESI-positive full scan/data-dependent tandem MS (MS/MS) (Table 1). It was shown that LIXI was completely metabolized with t_0.5_ < 0.5 h in all examined species. The other 3 examined peptides, i.e., EXE, LIRA, and SEMA, showed higher stability. In particular, LIRA and EXE were shown to be the most stable, with t_0.5_ higher than 4 h, followed by SEMA.

In the next study from the literature [27], the absorption, metabolism, and excretion of SEMA were investigated in healthy humans and compared with respective data from non-clinical studies. UPLC chromatography of the [3H]-radiolabeled metabolites from plasma, urine, and feces was conducted by collecting respective fractions and measuring radioactivity with a scintillation counter. The LC-MS analysis was carried out using a QTRAP (AB Sciex LLC, Framingham, MA, USA) mass spectrometer in the multiple reaction monitoring (MRM) mode. The main goal of this study was to investigate the metabolism of SEMA after a single subcutaneous injection of 0.5 mg [3H]-semaglutide in healthy adult men and to compare the results with those from non-clinical studies concerning SEMA. The parent drug was shown to be the main compound circulating in the plasma of humans as well as rats and monkeys. However, it was also metabolized with similar metabolite profiles within all these species. Six metabolites were identified in human plasma, among which the most abundant one accounted for 7.7% of total radioactivity. Similar to humans, 10 and 4 metabolites were identified in the plasma of rats and monkeys, respectively, each accounting for less than 9% of all radioactive material. At the same time, it was confirmed that the metabolites of SEMA were formed via proteolytic cleavage of the peptide backbone and beta-oxidation of the fatty acid side chain. All the metabolites in urine and feces showed shorter retention times than parent SEMA in the RP chromatography. Two metabolites in urine were identified as the free Lys26 amino acid with butyric (C4) and hexanoic (C6) diacid side chains, respectively [27].

### 3.2. Chemical Degradation of Glutides (GLP-1 RAs) and the Methods Used for Elucidate Their Degradation Pathways

Similarly to low-mass drug (small drug) molecules, regulatory agencies recommend characterizing the chemical stability and related impurities of peptide drugs. These impurities may occur as a result of degradation during manufacture or storage and decrease product efficacy or even exert some toxic effects. It is reported that some impurities derived from the degradation of peptide drugs can even provoke anaphylactic shock [18]. It is also obvious that impurities from degradation processes in peptide drugs should be controlled using the appropriate strategies [17].

In the study from the literature [35], a few analytical methods to examine the degradation of exenatide (EXE) in solutions of different pH were described. Firstly, EXE and its impurities were separated on a C4-Pack column using PDA detection and analyzed by a mass spectrometer with a Q-TOF with a dual ESI system. The total ion chromatogram (TIC) was obtained at 280 nm (Table 1). Secondly, impurities in EXE were separated using HPLC-SEC using a dedicated Superdex Increase 75 GL column from Cytiva (Marlborough, MA, USA) (10 × 300 mm) and recorded using an UV detector. Samples were separated with phosphate buffered saline as the mobile phase at a flow rate of 1.0 mL/min using isocratic elution. In addition, the intrinsic fluorescence of proteins was used to determine the tertiary structure of EXE. Analysis was performed at the wavelength range of 280–450 nm for emission and at wavelength 270 nm for excitation. Finally, the circular dichroism method was used to determine the secondary structure of EXE over a wavelength range of 245 to 195 nm [35]. 

It was shown that EXE is relatively stable at pH 4.5, while at pH > 4,5, considerable degradation of the drug was observed. It was concluded that EXE undergoes deamidation and Trp-cage unfolding that seem to precede further degradation, including dimerization, aggregation, and precipitation. Oxidation in the helical region of EXE was also observed, which could potentially lead to fragmentation and aggregation. When pH was in the range of 5.5–6.5, the Authors reported oxidation as the main mechanism of degradation. On the other hand, they suggested degradation of EXE via deamidation and aggregation in the pH range of 7.5–8.5. In conclusion, the Authors suggested the usefulness of these interesting results in the development of generic products of EXE as well as some novel drug delivery systems for EXE and similar drugs [35]. The LC-HRMS method was also developed by Zeng et al. [18] for three peptide drugs, including EXE, for monitoring the respective drug products quality. Separation of peptide degradants was performed using a Waters UPLC BEH C18 column using gradient elution (Table 1). The results obtained here proved that LC-HRMS methods are able to determine amino acid composition, confirm peptide sequences, and quantify impurities, even when they are co-eluted with each other.

An accurate, precise, and robust RP-HPLC method [36] was developed for the estimation of semaglutide (SEMA) using a Kromasil C18 column from Merck (4.6 × 250 mm, 5 µm), and the mobile phase consisted of phosphate buffer and MeOH in a ratio of 61.2:38.8. Detection was carried out at a wavelength of 230 nm. Stress degradation studies were performed for the drug under various stress conditions. No peaks of degradants were found in the cases of acidic and neutral hydrolysis, photodegradation, or thermal degradation. However, two degradants were found in the case of alkaline degradation and one in oxidative conditions. 

In the study of Zhang et al. [37], separation of the parent SEMA and its impurities, followed by determination of the molecular weight of these impurities, was reported using RP-UPLC-MS/MS. The main purpose of the mentioned study was to characterize the low-level D-amino acid isomeric impurities. Respective samples of SEMA went through lyophilization, hydrolyzation with deuterated HCl, derivatization with chiral low level D/L type amino acid shifting, and finally, analysis of different amino acids and comparison with respective D/L amino acid standards. As a result, the Authors reported an accurate and sensitive method for the low-level D-Ser8, D-His1, and D-Asp9 impurities of SEMA in respective SEMA formulations [37].

## 4. Gliflozins (SGLT2 Inhibitors)

Gliflozins are members of a group of relatively new SGLT2 inhibitors that increase glucosuria by inhibiting glucose reabsorption in the kidney. Since the approval of dapagliflozin (DAPA, (2S,3R,4R,5S,6R)-2-[4-chloro-3-[(4-ethoxyphenyl)methyl]phenyl]-6-(hydroxymethyl)oxane-3,4,5-triol) as the first SGLT2 inhibitor in 2012, many other drugs have been developed and approved, including canagliflozin (CANA, (2S,3R,4R,5S,6R)-2-[3-[[5-(4-fluorophenyl)thiophen-2-yl]methyl]-4-methylphenyl]-6-(hydroxymethyl)oxane-3,4,5-triol), empagliflozin (EMPA, (2S,3R,4R,5S,6R)-2-[4-chloro-3-[[4-[(3S)-oxolan-3-yl]oxyphenyl]methyl]phenyl]-6-(hydroxymethyl)oxane-3,4,5-triol), ertugliflozin (ERTU, (1S,2S,3S,4R,5S)-5-[4-chloro-3-[(4-ethoxyphenyl)methyl]phenyl]-1-(hydroxymethyl)-6,8-dioxabicyclo[3.2.1]octane-2,3,4-triol), ipragliflozin (IPRA, (2S,3R,4R,5S,6R)-2-[3-(1-benzothiophen-2-ylmethyl)-4-fluorophenyl]-6-(hydroxymethyl)oxane-3,4,5-triol), tofogliflozin (TOFO, (3S,3′R,4′S,5′S,6′R)-5-[(4-ethylphenyl)methyl]-6′-(hydroxymethyl)spiro [1H-2-benzofuran-3,2′-oxane]-3′,4′,5′-triol), luseogliflozin (LUSE, (2S,3R,4R,5S,6R)-2-[5-[(4-ethoxyphenyl)methyl]-2-methoxy-4-methylphenyl]-6-(hydroxy me thyl)thiane-3,4,5-triol) and bexagliflozin (BEXA, (2S,3R,4R,5S,6R)-2-[4-chloro-3-[[4-(2-cyclopropyloxyethoxy)phenyl]methyl]phenyl]-6-(hydroxymethyl)oxane-3,4,5-triol) [1].

**Figure 2:**
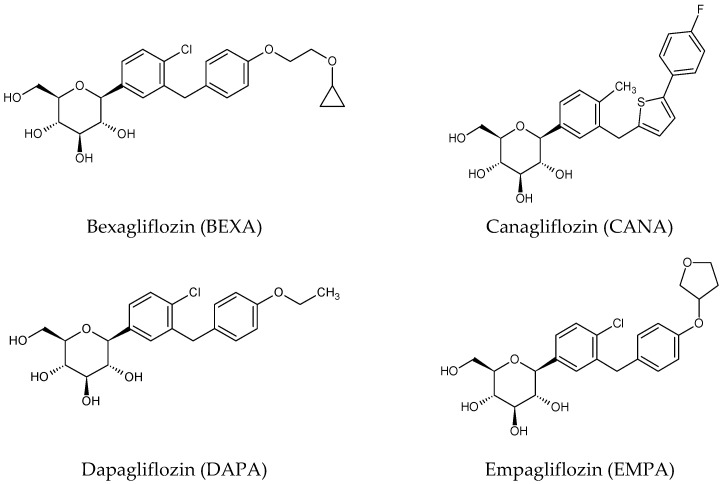

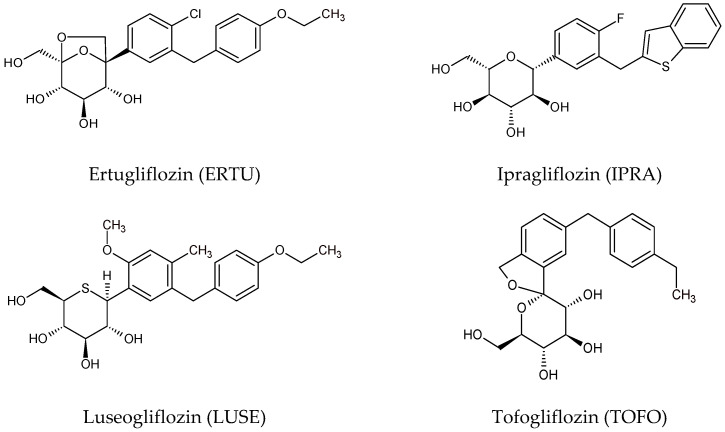
Chemical structures of SGLT2 inhibitors: bexagliflozin (BEXA), canagliflozin (CANA), dapagliflozin (DAPA), empagliflozin (EMPA), ertugliflozin (ERTU), ipragliflozin (IPRA), luseogliflozin (LUSE) and tofogliflozin (TOFO).

By targeting the kidney, gliflozins have a unique mechanism of action that results in enhanced glucosuria, osmotic diuresis, and natriuresis. Thereby, they improve glucose control with a limited risk of hypoglycemia. In addition, they have shown favorable effects on cardiovascular risk factors such as body weight, blood pressure, lipid profile, arterial stiffness, and endothelial functions [3,5]. The predominant pathophysiological mechanisms that may explain the cardiovascular benefits of SGLT2 include plasma volume and diuresis, cardiac fibrosis, myocardial metabolism, and adipokine kinetics. Therefore, the current guidelines propose a new procedure in the management of T2DM with a preferential place for SGLT2, even before metformin, especially in patients with atherosclerotic cardiovascular disease, heart failure, and progressive kidney disease [1]. As far as the gliflozin presence on the pharmaceutical market is concerned, DAPA was approved by EMA in 2012 and by FDA in 2014; CANA by both EMA and FDA in 2013; and EMPA in 2014. LUSE, IPRA, and TOFO were approved in Japan in 2014. ERTU was approved by the FDA in 2017 and by the EMA in 2018, and BEXA by the FDA in 2023. The structures of the mentioned gliflozins are presented in Figure 2.

### 4.1. Metabolic Transformations of Gliflozins (SGLT2 Inhibitors) and the Methods Used to Examine Their Metabolic Pathways

In the study of Zhang et al. [28], the samples from humans and animals receiving bexagliflozin (BEXA) were fractionated by an HPLC system and examined using a radioactivity flow monitor. Radioactivity in bulk samples was determined using a liquid scintillation analyzer. Quench correction was checked using quenched radioactive reference standards. In the next step, unlabeled rat plasma samples were analyzed using the next HPLC system with detection on a triple quadrupole mass spectrometer equipped with an atmospheric pressure chemical ionization (APCI) interface. For the analysis of unlabeled monkey samples, an LC-MS/MS mass spectrometer equipped with a turbo ion spray with an ESI interface was applied (Table 1).

**Figure 3:**
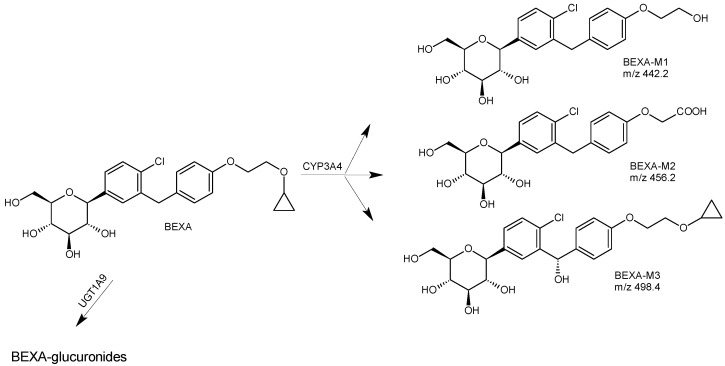
Main metabolic pathways of SGLT2 inhibitor bexagliflozin (BEXA) [28].

Based on the results obtained by the Authors, BEXA was shown to be metabolized via glucuronidation and oxidation to form six principal metabolites, i.e., three non-active metabolites through glucuronidation and three through oxidation, i.e., BEXA-M1, BEXA-M2, and BEXA-M3 (Figure 3). In vitro studies identified CYP3A4 and UDP-glucuronosyltransferase UGT1A9 as the major enzymes acting on the BEXA moiety. Following oral dosing of humans with [14C]-BEXA, the 3-O-glucuronide contributed 32% of the parent drug, and all other metabolites contributed < 10%. The input of recovered radioactivity was ca. 90%, and of this, ca. 50% was present in feces, predominantly as BEXA, and ca. 40% was present in urine, mainly as 3-O-glucuronide. At the same time, BEXA-M2 was shown as a major metabolite in vivo in all species examined. The pharmacological activity of these metabolites was assessed by measuring the sodium-dependent uptake of the particular substrate, methyl-a-D-[U-14C]-glucopyranoside, that was not metabolized, by cells expressing recombinant human SGLT2 protein. All metabolites had less than 10% of the activity of the parent BEXA [28].

In the study of Francke et al. [38], separation of canagliflozin (CANA) and its metabolites was achieved using a Waters XBridge RP-HPLC column (4.6 × 250 mm, 5 µm) at 25 °C. A flow rate of the mobile phase of 1 mL/min was used throughout the analysis. Sample components were eluted with a gradient elution consisting of solvent A (0.025 mM ammonium acetate, pH 9) and solvent B (10/45/45, 0.25 M ammonium acetate of pH 9/MeOH/ACN). The eluates from the HPLC column were steered to both a PDA detector and a radioactivity detector. Finally, the amount of the unchanged CANA was calculated from the radioactivity peaks. The main goal of this study was to examine the formation of CANA O-glucuronides in microsomes from human liver, intestinal, and kidney tissues using [14C]-CANA. Furthermore, the impact of genetic variations in UGTs on the pharmacokinetics of CANA was studied in detail [38].

Separation of glucuronides of CANA was also performed in the study of Algeelani et al. [39], using a HPLC method on a Waters μBondaPak C18 column (3.9 × 300 mm) with fluorescence detection. The elution was isocratic, with the mobile phase at pH 3.2 consisting of ACN and 20 mM phosphate buffer (55:45, *v*/*v*) with a flow rate of 1 mL/min. For detecting respective eluents, the wavelength 280 nm was used for excitation, and the wavelength 325 nm was used for emission.

**Figure 4:**
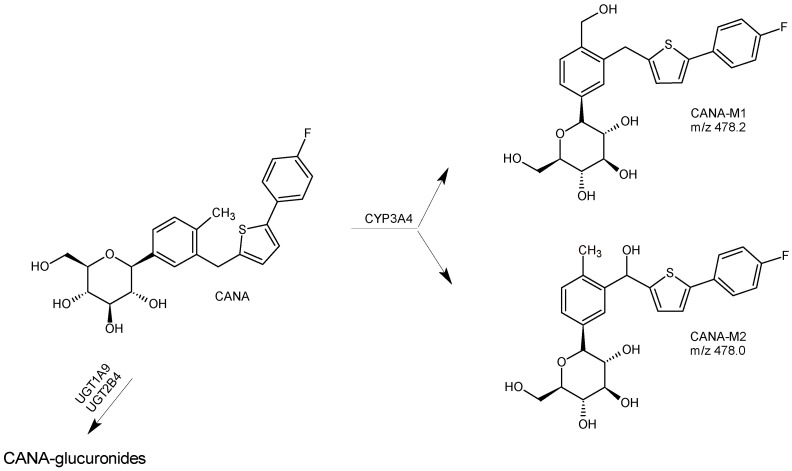
Main metabolic pathways of SGLT2 inhibitors canagliflozin (CANA) [29,40].

Reliable and selective quantification of CANA and its metabolites from human and rat samples was also developed using UPLC-TOF-MS/MS [29]. Following liquid-liquid extraction with tert-butyl methyl ether, separation was performed on a Waters XBridge BEH C18 column. Information-dependent acquisition (IDA) was used to obtain full-scan mass spectra and dependent product ion spectra (Table 1). It was confirmed that CANA was mainly metabolized via O-glucuronidation by the glucuronosultransferases UGT1A9 and UGT2B4, and to a lesser extent by CYP3A4 (CANA-M1). In rat plasma, other metabolites were detected, including CANA-M2 (Figure 4). However, their amounts were almost too low to quantify them using the above method [29]. In the study of De Jesus et al. [40], the above-mentioned structures for the mentioned CANA metabolites were confirmed.

**Figure 5:**
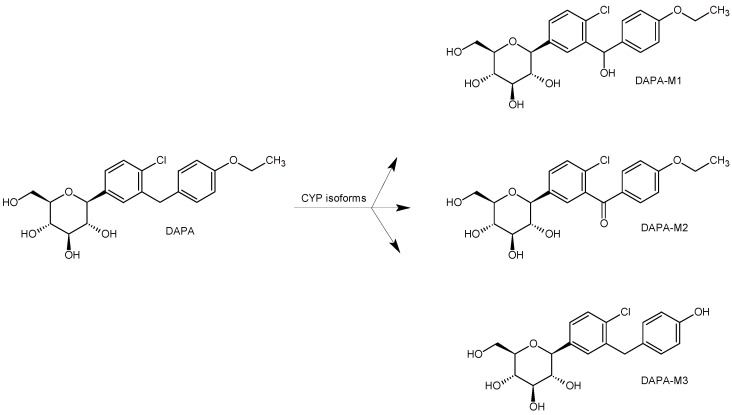
Main metabolic pathways of SGLT2 inhibitor dapagliflozin (DAPA) [31].

In addition, LC-MS experiments were performed to study DAPA metabolism (Table 1), and a few products of metabolism were proposed, i.e., DAPA-M1-DAPA-M3 (Figure 5) [31].

In the study of Lapham et al. [41], the metabolism of ertugliflozin (ERTU) through various UGT and CYP enzyme-mediated pathways was examined. The Authors discussed the choice of particular enzymes and chemical inhibitors for these metabolism studies. It was stated that the metabolism of ERTU in human liver microsomes mainly occurred via glucuronidation and UGT1A (78%) or UGT2B7/UGT2B4 (18%), and via oxidation and CYP3A4 (3.4%) or CYP2C8 (0.16%). Bearing in mind these results as well as the data from the literature, the Authors concluded that human systemic clearance of ERTU could be mediated by UGT1A9 (70%), UGT2B7/UGT2B4 (16%), CYP3A4 (10%), CYP3A5 (1.2%), CYP2C8 (0.5%), and renal elimination (2%).

**Figure 6:**
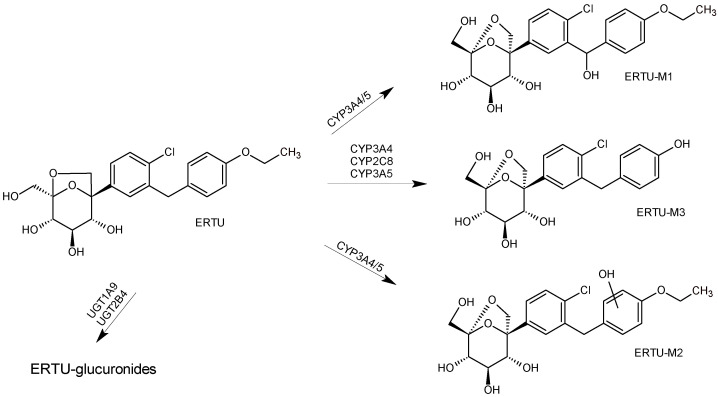
Main metabolic pathways of SGLT2 inhibitor ertugliflozin (ERTU) [41].

Based on a mass-balance study, the major metabolites were described as 3-O-and 2-O-glucuronides. As stated above, the oxidative metabolism of ERTU was minor, but a few oxidative metabolites, i.e., monohydroxylated ERTU-M1 and ERTU-M2, and des-ethyl ERTU-M3, were also proposed (Figure 6).

**Figure 7:**
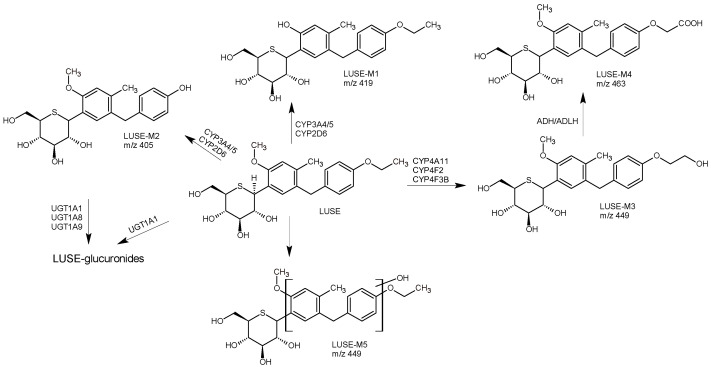
Metabolic pathways of luesogliflozin in humans (LUSE); *m*/*z* [M-H]^−^ [42].

In the study of Miyata et al. [42], the primary metabolic pathways of luseogliflozin (LUSE) and the enzymes responsible for these transformations were investigated in humans. It was shown that, in plasma, the unchanged parent LUSE gave the most abundant peak. However, over 10 minor metabolites were observed. The primary metabolic pathways involved O-demethylation of the unchanged drug followed by hydroxylation of the 5-benzyl-4-methylphenylmoiety to form LUSE-M1, O-deethylation to form LUSE-M2, and subsequent glucuronidation; ω-hydroxylation at the ethoxy group to form LUSE-M3 followed by oxidation to form the corresponding carboxylic acid metabolite LUSE-M4, as well as direct glucuronidation. Further studies indicated that the formation of LUSE-M2 was mainly mediated by CYP3A4/5, and subsequently, glucuronidation was catalyzed by UGT1A1, UGT1A8, and UGT1A9. The formation of LUSE-M3 was mediated by CYP4A11, CYP4F2, and CYP4F3B, while further oxidation of LUSE-M3 to LUSE-M5 was mediated by alcohol dehydrogenase and aldehyde dehydrogenase. These results demonstrated that LUSE is metabolized through multiple pathways, including CYP-mediated oxidation and UGT-mediated glucuronidation (Figure 7) [42].

For simultaneous determination of the above-mentioned LUSE metabolites in humans, plasma and urine samples were spiked with deuterated internal standards and subjected to SPE [43]. Furthermore, chromatographic separation was performed on a YMC-Pack Pro C18 analytical column from YMC Co., Ltd. (Kyoto, Japan) with 0.1% CH_3_COOH in H_2_O and ACN under gradient elution. A mass spectrometer with a turbo ion spray with an ESI interface in a negative ionization mode was used. This study was conducted to evaluate the metabolism of LUSE in patients with normal renal functions or with various degrees of renal impairment. Finally, no differences were observed between both groups of patients. Consequently, it appeared that renal impairment has no significant effect on the pharmacokinetics of LUSE [43].

In the study of Zell et al. [44], the metabolism pathway and quantitative determination of tofogliflozin (TOFO) and its metabolites were evaluated following administration of a single oral dose of [14C]-TOFO to six healthy subjects. The column-switching HPLC system consisted of the trapping column YMC-Pack ODS-AQ column (2.1 × 20 mm, 5 µm) and the analytical column Discovery HS (2.1 × 50 mm, 3 µm) from Merck. Gradient elution was performed with a mixture of 10 mM ammonium acetate and 5 mM ammonium acetate/98% HCOOH/MeOH (10/0.02/90; *v*/*v*/*v*).

The ODS-AQ used by the Authors is a unique RP material that presents both hydrophobic properties due to high carbon loading as well as hydrophylic properties because of a relatively hydrophilic surface. Due to this hydrophylic surface, ODS-AQ can be “wetted” with polar eluents, resulting in good peak shape in highly aqueous mobile phases. However, in such highly aqueous mobile phases, the above ODS-AQ packing can give longer retention times of the analytes than conventional ODS packing. This is due to the C18 ligands on ODS-AQ being lifted from the surface by the action of the eluents penetrating the silica pores of the packing [44].

The same HPLC method was used for mass spectrometry with a QTRAP instrument for the identification of TOFO metabolites. IDA was used to obtain full-scan mass spectra and dependent product ion spectra. Enhanced mass scan mode (EMS) was used as the survey scan, which is the full scan mode where all ions in the specified *m*/*z* range are trapped in quadrupole Q3 prior to detection. Product ion data were collected using an enhanced product ion scan (EPI) triggered by an intensity threshold of the EMS signal [44].

**Figure 8:**
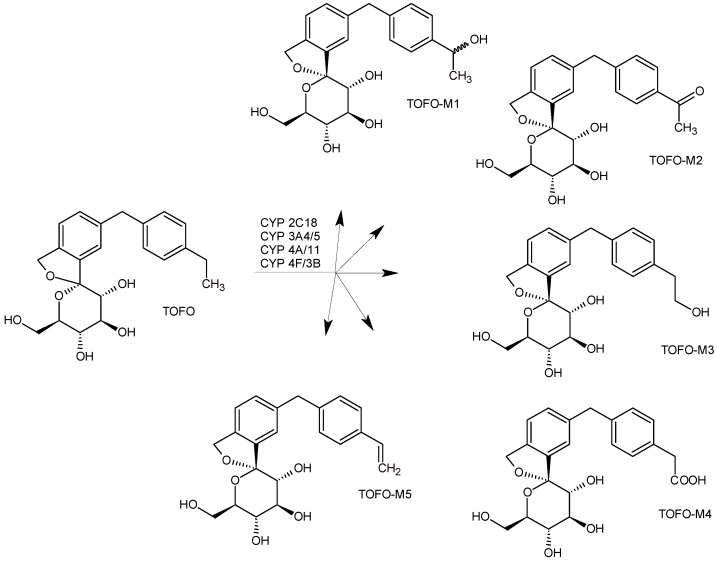
Metabolic pathways of tofogliflozin in humans (TOFO) [44,45].

The above column-switching system and the same HPLC conditions were used for effluent fractionation to obtain the reconstructed radiochromatograms for metabolite profiling. The effluent from the analytical column was then collected into a 384-well microplate using a MALDI spotter. Following evaporation of the solvent, the plate was subjected to solid scintillation counting (SSC). As a result, the metabolic pathway of TOFO exemplified the “soft spot” in the parent drug molecule, i.e., the ethyl phenyl moiety, which was prone to phase I of metabolism. The hydroxyl metabolite TOFO-M1 underwent further oxidation to the respective ketone metabolite TOFO-M2. The next hydroxyl metabolite, TOFO-M3, was further oxidized to the corresponding phenyl acetic acid metabolite, TOFO-M4. Next, TOFO-M4 and also the parent drug were shown to form glucuronides, but no other conjugates of phase II metabolism. In addition, TOFO-M2 and the pharmacologically active TOFO-M5 metabolites (Figure 8) can be produced from the parent drug via dehydrogenation catalyzed by CYP3A. However, because of their very low occurrence in plasma, the active TOFO-M5 was not examined quantitatively by the Authors [44].

In the study of Schwab et al. [45], the study to investigate the pharmacokinetics, metabolism, and excretion of TOFO was conducted after a single oral dose of [14C]-TOFO and an intravenous microdose of [13C]-TOFO in healthy male subjects.

A microdose is defined as a dose of up to 100 µg of the respective substance per subject. A lot of information as well as many different objectives can be attained through the use of microdosing. The main advantage of such a procedure is an assessment of the pharmacokinetics and metabolism of particular compounds following both oral and intravenous administration and the possibility of determining the absolute drug bioavailability. The last could be studied by adding a concomitant intravenous isotope tracer to a pharmacological dose of the drug together with the non-labeled drug that is administered orally. The choice of this intravenously administered tracer is typically a [14C]-label, which is quantified by the AMS procedure after proper separation [45].

Quantification of [12C]-TOFO in plasma and urine and [13C]-TOFO in plasma was also performed by selective LC-MS/MS methods [45]. Quantification of [13C]-TOFO was performed using the off-line SPE procedure on OASIS HLB l elution plates from Waters, followed by microelution as the first preparation step. Furthermore, an on-line dilution with subsequent on-line SPE as a part of the column switching system was used. MS detection of the analytes and respective internal standards was performed in SRM in the positive ion mode on a triple quadrupole mass spectrometer. The [14C]-tracer was administered orally and used to quantify the total drug-related circulating radioactivity. In addition, the time course and recovery of total radioactivity in the excreta were determined using this tracer. Furthermore, the [13C]-tracer was administered intravenously as a concomitant to the oral administration and used for quantification of bioavailability and some kinetic parameters of the drug. It was shown that the positions of the 13C and 14C labels in the TOFO structure need to be in a metabolically inert region of the molecule to ensure proper metabolite tracking. This goal was achieved in the present study by the introduction of a hexa-[13C]phenyl moiety and a single [14C]-atom into the side chain [45]. Following oral administration of 20 mg of TOFO, the carboxylate metabolite TOFO-M4 was the major circulating metabolite, while the ketone metabolite TOFO-M2 represented the minor circulating one. The sum of the area under the plasma concentration-time curve from time 0 to 24 h (AUC 24) of TOFO, TOFO-M4, and TOFO-M2 accounted for ca. 99% of the AUC 24 of total radioactivity. The main metabolites excreted into urine were TOFO-M4 and the parent TOFO, followed by a minor amount of TOFO-M2 [45].

### 4.2. Chemical Degradation of Gliflozins (SGLT2 Inhibitors) and the Methods Used for Elucidate Their Degradation Pathways

**Figure 9:**
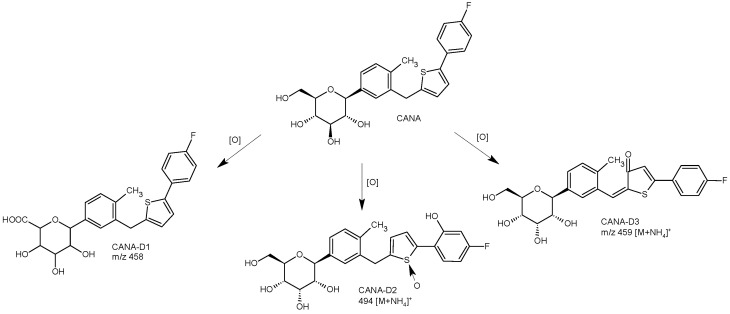
Degradation pathways of canagliflozin (CANA) [30,46].

In the study of Emam and Abdelwaham [46], CANA was quantified along with its oxidative degradation product by an eco-friendly HPLC method. Separation and quantitation were performed on a Zorbax Eclipse C18 column from Agilent Technologies (4.6 × 250 mm, 5 μm) with the mobile phase consisting of MeOH-H_2_O (98:2, *v*/*v*) at a flow rate of 1 mL/min and UV detection at 225 nm. The column temperature was maintained at 25 °C. The main oxidative degradation product was obtained by refluxing CANA with 3% hydrogen peroxide at high temperatures. The structure of the prepared degradation product showed the presence of a carboxylic group (CANA-D1) (Figure 9) that was confirmed using IR analysis [46].

The degradation processes of CANA were also studied using UPLC and LC/ESI-QTOF-MS/MS analysis [30]. Separation of the drug and its degradants was monitored at 291 nm. The same UPLC conditions were applied for LC/HRMS analysis (Table 1). Degradation of CANA was observed under oxidative and acidic stress conditions, whereas it was stable under base and neutral hydrolysis as well as photolytic and thermal stress. The oxidative degradants were identified as CANA-D2 and CANA-D3 (Figure 9). Formation of CANA-D2 can be explained by S-oxidation of the thiophene ring and hydroxylation of the fluorobenzene ring of the parent drug, bearing in mind that thiophene sulfur is rather prone to oxidation. The elemental compositions of CANA-D2 and its product ions in MS were confirmed by accurate mass measurements. Based on these data, CANA-D2 was identified as (2S,3R,4S,5S,6R)-2-(3-((5-(4-fluoro-2-hydroxyphenyl)thiophen-1-oxide-2-yl)methyl)-4-methylphenyl)-6-(hydroxymethyl)tetrahydro-2H-pyran-3,4,5-triol. As far as CANA-D3 is concerned, the addition of an oxygen atom on the thiophene ring and the formation of 5-(4-fluorophenyl)-2-methylenethiophen-3(2H)-one were suggested. Based on these data, the name for CANA-D3 was proposed as (E)-5-(4-fluorophenyl)-2-(2-methyl-5-((2S,3R,4S,5S,6R)-3,4,5-trihydroxy-6-(hydroxymethyl)tetrahydro-2Hpyran-2-yl)benzylidene)thiophen-3(2H)-one [30].

Finally, the toxicity of CANA and its degradants was evaluated using computational tools, i.e., TOPKAT (Discovery Studio 2.5, Accelrys Inc., San Diego, CA. USA) and DEREK (Lhasa Limited, Leeds, UK). TOPKAT software assesses toxicological endpoints based on probability values by using quantitative structure-toxicity relationship models, while DEREK uses rules derived from existing literature. These rules are based on knowledge of structural alert-toxicity relationships. By using these calculations, similar low toxicity profiles of CANA and its degradants were shown, probably due to the presence of the pyrantriol moiety in all four molecules. However, it was speculated that CANA-D1 exhibits a high probability of ocular irritancy [30].

**Figure 10:**
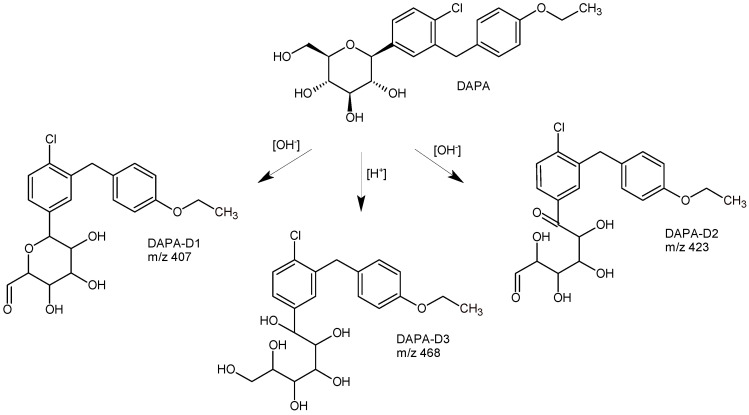
Degradation pathways of dapagliflozin (DAPA) [31].

The next paper from the literature [31] describes a LC-MS method for analyzing DAPA and its potent degradants on a Zorbax Eclipse C8 column from Agilent Technologies (Table 1). The degradation products, i.e., DAPA-D1 and DAPA-D2, were formed under basic conditions, while DAPA-D3 was formed after acid degradation (Figure 10). At the same time, DAPA was found to be stable at dry heat and, similarly, during photolytic stress. It was proposed that DAPA-D3 is formed from DAPA by the attack of H+ on the oxygen of the oxane ring, which leads to the oxane ring opening in acidic conditions. The remaining degradants, i.e., DAPA-D1 and DAPA-D2, could be formed by the attack of OH^-^, leading to oxane ring opening in alkaline conditions [31].

In silico toxicity prediction of DAPA and its degradation products was also performed with ProTox-II software (https:/tox.charite.de) that classified toxicity with different targets, such as oral, hepatic, carcinogenic, immunotoxicity, mutagenicity, and cytotoxicity. Various Tox21-Nuclear receptor signaling pathways, Tox21-stress response pathways, and toxicity targets were included in this study. Prediction tools identified that there is no binding of DAPA and its degradation products to any of the toxicity targets, and similarly, most of the toxicity end points and pathways are inactive. However, immunotoxicity end point and aryl hydrocarbon receptor signaling pathways are active in the case of DAPA-D2, and mitochondrial membrane potential stress response pathways are active in the cases of DAPA-D2 and DAPA-D1 [47].

The next study from the literature [48] confirmed that DAPA is susceptible to acidic, alkaline, and oxidative degradation but resistant to UV light. In addition, the impact of metformin as a concomitant molecule was studied. Separation of DAPA and their degradants derived from these accelerated degradations was carried out using a Thermo Fisher Symmetry Acclaim C18 column (2.1 × 100 mm, 2.2 μm) via isocratic elution. The mixture of 0.05 M phosphate buffer at pH 3.5 and ACN (50:50, *v*/*v*) was used as the mobile phase with a flow rate of 0.4 mL/min. A wavelength of 225 nm was selected for the determination of both DAPA and metformin, while the temperature of the column oven was maintained at 60 °C. Finally, the degradation pathway of DAPA in acidic, alkaline, and oxidative conditions was suggested as a result of the cleavage of the sugar part of the glycoside from the parent structure, which was reported for the first time.

In the study of Vichare et al. [49], the stability of EMPA in the presence of metformin was examined using high performance thin-layer chromatography (HPTLC). A system with pre-coated silica gel plates, a twin-trough chamber, and a TLC Scanner were used. In addition, LC-MS/MS studies were completed using a HPLC system with a Zorbax Eclipse Plus C18 column from Agilent Technologies (2.1 × 50 mm, 1.8 μm) and a Q-TOF mass spectrometer with positive ESI mode.

**Figure 11:**
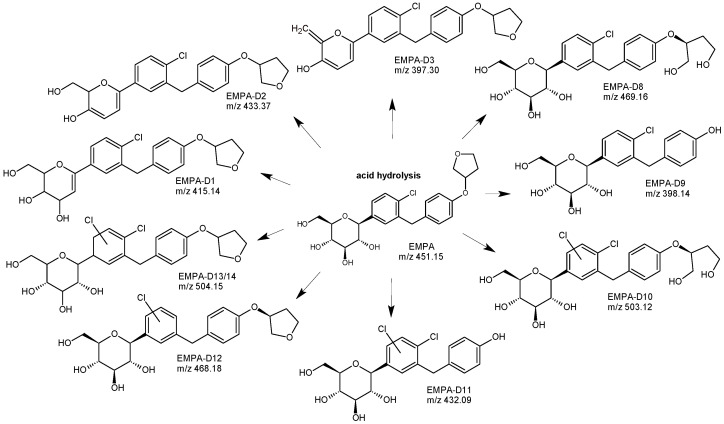
Degradation pathways of empagliflozin (EMPA) in acidic conditions; for EMPA-D8-EMPA-D14 *m*/*z* [M+NH_4_]^+^ [32,33,49].

**Figure 12:**
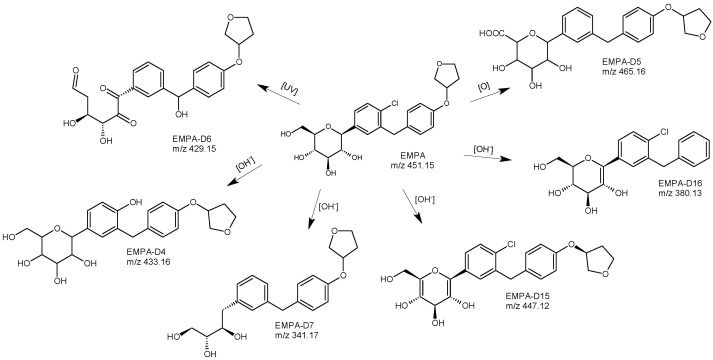
Degradation pathways of empagliflozin (EMPA) in alkaline, oxidative and photolytic conditions; for EMPA-D15 and EMPA-D16 *m*/*z* [M+NH_4_]^+^ [32,33,49].

Forced degradation studies were performed under different stress conditions, and then the degraded samples were analyzed by the developed method. In acidic conditions EMPA, showed 28.76% degradation with the formation of 3 degradants, namely, EMPA-D1, EMPA-D2, and EMPA-D3 (Figure 11). Based on LC-MS/MS studies, the probable structures of these degradants were proposed. EMPA-D1 was identified as 6-(4-chloro-3-{[4-(oxalan-3yloxy)phenyl]methyl}phenyl)-2-(hydroxymethyl)-3,4-dihydro-2H-pyran-3,4-diol. The structure of 6-(4-chloro-3{[4-(oxalan-3 yloxy)phenyl]methyl}phenyl)-2-(hydroxymethyl)-2H-pyran-3-ol was proposed for EMPA-D2 while EMPA-D3 was specified as 6-(4-chloro-3{[4-(oxalan-3-yloxy)phenyl]methyl}phenyl)-2-methylidene-2H-pyran-3-ol. Under alkaline conditions, EMPA showed degradation of 15.48%, and the formation of the next 2 degradants was reported. From the LC-MS/MS data, the probable structure of one of them, i.e., EMPA-D4 was proposed as 2-(4-hydroxy-3{[4-(oxalan-3-yloxy)phenyl]methyl}phenyl)-6-(hydroxymethyl)-oxane-3,4,5-triol). By analyzing the obtained spectra, the substitution of the chlorine atom by the OH group was proposed. Under oxidative conditions, EMPA was found to generate one more degradant, i.e., EMPA-D5 (Figure 12), that could be formed by oxidation of the primary alcoholic group to carboxylic acid to form 6-(4-chloro-3{[4-(oxalan-3-yloxy)phenyl]methyl}phenyl)-3,4,5-trihydroxyoxane-2-carboxylic acid [49].

In the next study concerning EMPA [32], a simple and fast analytical method with a low limit of quantification was developed and validated to determine EMPA, its synthetic impurities, and its degradants. The LC method development and forced degradation were completed using the UHPLC-Q-TOF-MS system. The analyses were carried out using a Zorbax Eclipse Plus C18 column from Agilent. The Q-TOF system was operated in a broadband collision-induced dissociation (bbCID) acquisition mode (Table 1). Degradation samples from acidic, basic, and oxidative conditions, as well as from temperature and radiation (UVC) stress, were examined. As a result, chromatograms indicated degradation of EMPA exposed to UVC and base, showing the formation of two new degradation products. It was interesting to observe that both of them, i.e., EMPA-D6 and EMPA-D7 showed breaks in the glucose moiety.

The LC-UV method suitable for elucidating degradation of EMPA was also proposed by Niguram et al. [33] using a Kromasil 100 C18 column from Merck and UV detection at 226 nm (Table 1). The sum of degradants generated during accelerated degradation was resolved by the UHPLC-MS method. In acid hydrolysis, the drug showed eight degradation products (EMPA-D8-D14). However, in base hydrolytic conditions, only 2 degradants (EMPA-D15 and 16) (Figure 11) were observed, which were eluted after the parent drug peak and therefore indicated their less polar nature. In acidic conditions, the diol formation after the opening of the tetrahydrofuran ring was proposed to get the structure of EMPA-D8. It was identified as (2S,3R,4R,5S,6R)-2-(4-chloro-3-(4-(((S)-1,4-dihydroxybutan-2-yl)oxy)benzyl)phenyl)-6-(hydroxymethyl)tetrahydro-2H-pyran-3,4,5-triol. EMPA-D9 was formed due to the strong acid conditions where the ether linkage between the phenyl group and tetrahydrofuran ring was split, releasing the tetrahydrofuran moiety. As a result, the product was distinguished as (2S,3R,4R,5S,6R)-2-(4-chloro-3-(4-hydroxybenzyl) phenyl)-6-(hydroxymethyl)tetrahydro-2H-pyran-3,4,5-triol. The structure of EMPA-D10 was proposed as the result of the addition of a chlorine atom to the phenyl ring attached to the sugar moiety and, subsequently, the opening of the tetrahydrofuran ring to form the respective diol. In the presence of strong acid, EMPA was also shown to breakdown the tetrahydrofuran moiety and chlorination on the phenyl ring, forming EMPA-D11. For EMPA-D12, it was proposed that it could be a positional isomer of the parent drug, possessing a shift in the site of the chlorine atom. For the next two degradants, i.e., EMPA-D13 and 14, the addition of a chlorine atom with the saturation of a double bond was proposed. These two degradants could be positional isomers or diastereomers of each other. Base hydrolysis of the parent drug caused the next 2 degradants, i.e., EMPA-D15 and D16 (Figure 12). The structure of EMPA-D15 might be due to the additional unsaturations in the structure of the parent drug molecule. In conclusion, the maximum degradants of EMPA were formed during acid hydrolysis, and the major ones were EMPA-D8 and EMPA-D9, generated due to the tetrafuran ring opening and elimination of the tetrahydrofuran ring, respectively [33].

**Figure 13:**
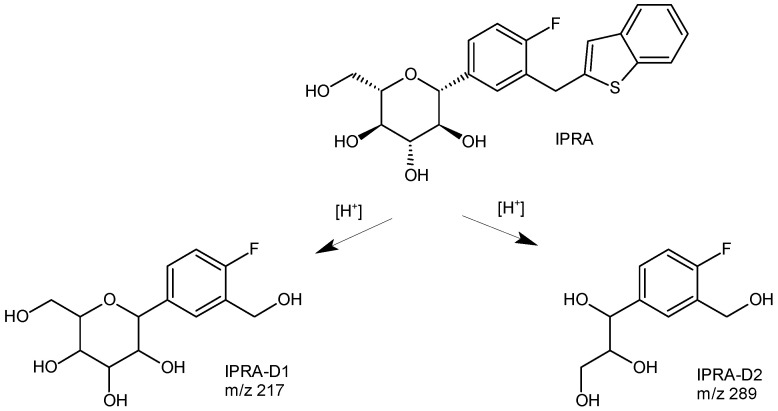
Degradation pathways of ipragliflozin (IPRA) in acidic conditions [34].

In the study of Elhassan et al. [34], IPRA degradation was studied under different stress conditions, i.e., acidic, basic, photolytic, oxidative, and thermal. These forced degradation experiments showed extensive degradation of IPRA under acidic, basic, and oxidative conditions, while high stability of IPRA under thermal and photolytic conditions occurred. Separation of the parent drug and respective degradation products was carried out using a Hypersil Gold UPLC C18 column from Thermo Fisher, while UV detection was performed at a wavelength of 230 nm (Table 1). The structural elucidation of the major degradation product (IPRA-D1) (Figure 13) showed the detachment of benzothiophene, producing 2-[4-fluoro-3-(hydroxymethyl)phenyl]-6-(hydroxyl methyl)oxane-3,4,5-triol. In the mass spectrometer, the cleavage of the sugar part of the glycoside by McLafferty rearrangement took place, giving the 1-propene-1,3-diol moiety and 1-[4-fluoro-3-(hydroxymethyl)phenyl]propane-1,2,3-triol (IPRA-D2) (Figure 13) [34]. As was shown above, the cleavage of the sugar part of the glycoside was also shown above for DAPA [48].

## 5. Conclusions

The main analytical tools for the analysis of glutides and gliflozins are undoubtely LC-MS or LC-HRMS methods. LC-HRMS is especially important for peptide drugs such as glutides. Mass spectrometers are frequently combined with a quadrupole such as a Q-TOF system, an ion trap, or orbitrap mass analyzers that deliver excellent resolution and mass accuracy, which are essential in drug metabolism and drug degradation studies [15]. In addition, radiometric methods using stable isotopes as well as chromatographic methods with deuterated standards could be used for the determination of the mentioned drugs in biological fluids or excreta. As was described above, [14C] and [13C] technology was widely applied for tracking glutides as well as gliflozins and providing their metabolic profiles.

**Figure 14:**
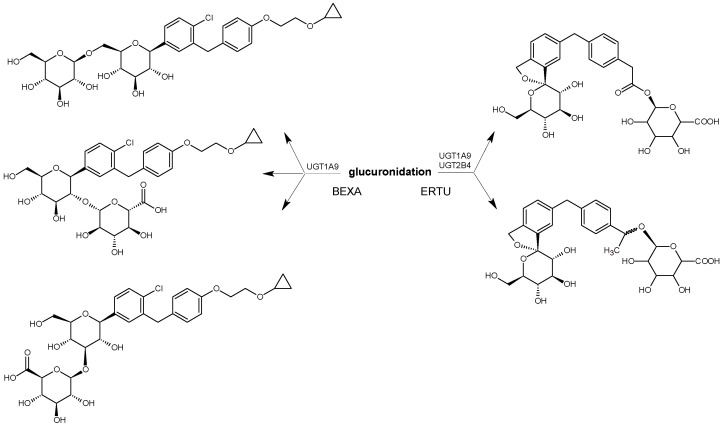
Typical products of metabolic transformations of bexagliflozin (BEXA) and ertugliflozin (ERTU) by glucuronidation [28,41].

It was also demonstrated that several SGLT2 inhibitors, including BEXA, CANA, DAPA, EMPA, and ERTU, share a similar metabolic pathway in humans and animals, primarily through O-glucuronidation. Their major non-active metabolites were identified as 1-O, 2-O, and 3-O-glucuronides (Figure 14). These SGLT2 inhibitors that metabolize through glucuronidation can be subsequently eliminated with urine. Such an excretion process can be facilitated by organic anion transporters, which are responsible for the uptake of various organic anionic compounds such as glucuronidation and sulfation drug metabolites [40].

**Figure 15:**
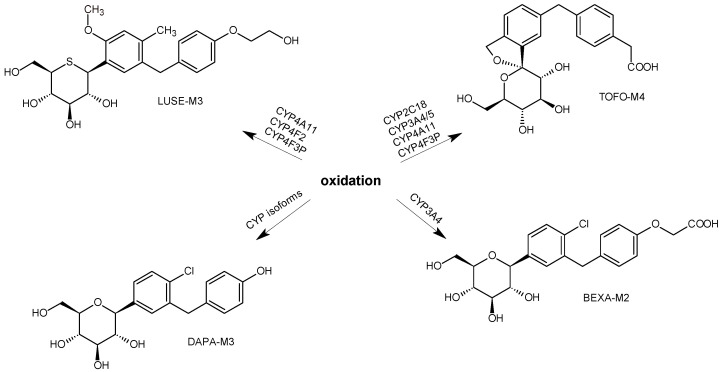
Metabolic transformations of SGLT2 inhibitors via oxidation: metabolites of bexagliflozin (BEXA-M2) [28], dapagliflozin (DAPA-M3) [31], luseogliflozin (LUSE-M3) [42] and tofogliflozin (TOFO-M4) [44].

**Figure 16:**
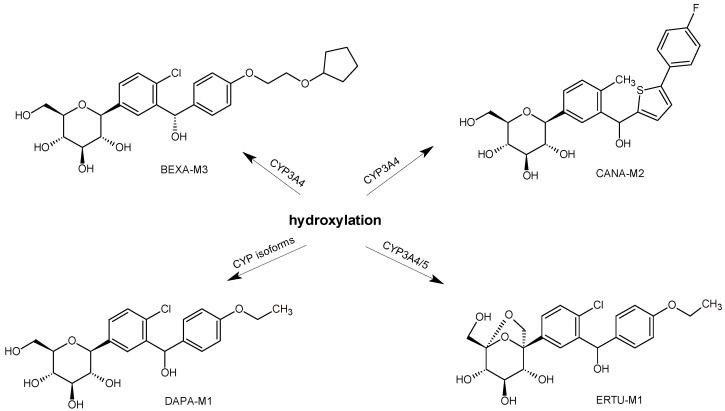
Metabolic transformations of SGLT2 inhibitors via hydroxylation: metabolites of bexagliflozin (BEXA-M3) [28], dapagliflozin (DAPA-M1) [31], canagliflozin (CANA-M2) [29] and ertugliflozin (ERTU-M1) [41].

Furthermore, the more or less important metabolic pathway of SGLT2 inhibitors via oxidative reactions occurred, e.g., for BEXA, DAPA, ERTU, LUSE, and TOFO. Such metabolic pathways, e.g., ω-hydroxylation at the ethoxy group followed by oxidation to form the corresponding carboxylic acid, were shown for LUSE, leading to the formation of LUSE-M4. The metabolic pathway via oxidation of the substituents at the 4-phenyl position of BEXA, DAPA, ERTU, LUSE, and TOFO seems to also be the characteristic pattern of their metabolism. The hydroxyl metabolite TOFO-M1 undergoes further oxidation to the respective ketone metabolite TOFO-M2. The next hydroxyl metabolite, TOFO-M3, is further oxidized to the corresponding phenyl acetic acid metabolite, TOFO-M4 (Figure 15). Furthermore, the process of hydroxylation of the benzyl-phenyl or benzyl-thiophen moieties is characteristic for BEXA, CANA, DAPA i ERTU (Figure 16).

Generally, both drug metabolism and drug degradation (during drug manufacturing and/or storage) can undergo similar chemical transformations. Consequently, many impurities that are generated during degradation could also be respective metabolites. Sometimes, individual metabolites as well as particular degradation products are formed or detected in small amounts, potentially with no toxicity risk. However, the goal of the present paper was to find as many such overlapping products of metabolism and chemical degradation of gliflozins as possible Thus, the metabolites and degradants formed in smaller amounts were also included. Further studies on possible degradation products of gliflozins based on their metabolic pathways should give a definitive assessment of their safety for patients. As far as such detected similarities between metabolites and degradants are concerned, the process of hydroxylation of the benzyl-phenyl or benzyl-thiophen moieties that was characteristic for metabolites of BEXA-M3, CANA-M2, DAPA-M1 and ERTU-M1 was observed during degradation of EMPA to form EMPA-D6.

## Figures and Tables

**Table 1:** LC-MS conditions for determination of metabolites and stress degradation products of glutides: exenatide (EXE), liraglutide (LIRA), lixiglutide (LIXI) and semaglutide (SEMA), and gliflozins: bexagliflozin (BEXA), canagliflozin (CANA), dapagliflozin (DAPA), empagliflozin (EMPA) and ipragliflozin (IPRA).

Compound	Conditions	Ref.
EXE LIRA LIXI SEMA	Waters XSelect CSH C18 XP column (2.1 × 50 mm, 2.5 µm) or Waters UPLC Peptide CSH C18 column (2.1 mm × 50 mm, 1.7 µm) and gradient elution with (A) 0.1% HCOOH in H_2_O and (B) 0.1% HCOOH in ACN, 0.4 mL/min; column temperature 60 °C. Orbitrap mass spectrometer operating in ESI positive full scan/data-dependent MS/MS.	[26]
EXE	LC-PDA: C4-Pack column and gradient elution with mobile phases consisted of ACN with 0.1% TFA and H_2_O with 0.1% TFA, 1.0 mL/min. LC-MS-Q-TOF: C4-Pack column and gradient elution using a mobile phase of ACN with 0.05% TFA and H_2_O with 0.05% TFA, 0.3 mL/min. Positive ESI: capillary voltage 1.2–2.0 kV, a drying gas temperature of 325 °C, a drying gas flow rate of 12 L/min, a nebulizer pressure of 45 psi and a fragmentor voltage of 225 V.	[27]
EXE	UPLC: Waters BEH C18 column (2.1 × 100 mm, 1.7 μm) and gradient elution with 0.1% HCOOH in H_2_O and 0.1% HCOOH in ACN, 0.3 mL/min; column temperature 30 °C, autosampler temperature 4 °C. Positive ESI: source gas temperature 350 °C, Vcap at 4000 V, fragmentor voltage at 210 V, skimmer 1 at 80.0 V, and octopole RF peak at 750 V.	[18]
BEXA	Triple quadrupole-APCI: Capcell Pak C18 MG column from Osaka Soda (Osaka, Japan) (4.6 × 100 mm, 5 µm) and isocratic elution with ACN: MeOH:H_2_O:10 mM ammonium acetate; 0.7 mL/min, MRM mode, collision energy 19, 22 or 37 eV. LC-MS/MS with turbo ion spray (ESI): Waters Nova-Pack VR C18 column (3.9 × 150 mm, 4 µm) and gradient elution with (A) 0.1% HCOOH in 0.5 mM ammonium acetate under positive and 5 mM ammonium acetate under negative mode, and (B) 0.1% HCOOH in mixture of ACN/MeOH (50/50), 0.8 mL/min. The ESI conditions: 4500 or 5500 V spray voltage, source temperature 550 °C, sheath gas N_2_, gas flow 60 psi; MRM mode; collision energy 37, 38, 45 or 47 eV. UPLC-Q-TOF: Waters ACQUITY HSS T3 C18 column (2.1 × 100 mm, 1.7 µm) and gradient elution with (A) 10 mM ammonium acetate and (B) ACN with 0.05% HCOOH, 0.5 mL/min; column temperature 40 °C; positive or negative ESI: source temperature 120 °C, the desolvation gas temperature 400 °C, the capillary voltage 3.0 kV and the cone voltage 20 V; desolvation gas N_2_ and the collision gas Ar.	[28]
CANA	UPLC-TOF-MS/MS: Waters XBridge BEH C18 column (2.1 × 100 mm, 2.5 µm) using 0.1% ACN-HCOOH (75:15, *v*/*v*), 0.7 mL/min; positive ESI: ion spray voltage, 5500 V; turbo spray temperature, 550 °C; declustering potential, 100 V; collision energy, 35 eV; N_2_ as the nebulizer and the auxiliary gas, and the nebulizer gas (gas 1), the heater gas (gas 2), and the curtain gas 55, 55, and 35 psi; collision energy, 35 eV; collision energy scope, 15 eV.	[29]
CANA	LC-PDA: Waters Acquity CSH C18 column (2.1 × 100 mm, 1.7 μm) with gradient elution using ACN-MeOH (70:30, *v*/*v*) and HCOOH; UV 291 nm. LC/ESI-Q-TOF-MS/MS: positive ESI with capillary voltage, 4000 V; fragmentor voltage, 150V; skimmer, 65 V; collision energy, 15–30 eV; drying gas, 320 °C, 10 L/min; nebulizing gas, 35 psi; N_2_ as drying gas, nebulizing gas and collision gas.	[30]
DAPA	LC-PDA: Zorbax Eclipse C8 column from Agilent Technologies (Santa Clara, CA, USA) (4.6 × 150 mm, 5 μm) and gradient elution with (A) 10 mM ammonium formate of pH 4 and (B) ACN, 1.0 mL/min; column temperature 30 °C and autosampler temperature 25 °C, UV 237 nm. Q-TOF MS: fragmentor voltage, 170 V; 3500 V; skimmer, 60 V; the drying gas at 350 °C, 10 L/min; N_2_ nebulizing gas 45 psi; MS^n^ studies: capillary voltage, 15–20 V; capillary temperature, 200 °C; spray voltage, 5 kV; tube lens offset voltage, 20 V; sheath gas N_2_, 30 psi pressure, and damping gas He; the 20–35 eV collision energies with 30 ms excitation time for CID.	[31]
EMPA	UHPLC-Q-TOF-MS: Zorbax Eclipse Plus C18 column from Agilent Technologies (2.1 × 50 mm, 1.8 µm) and mobile phase consisted of ACN and H_2_O Q-TOF-MS: positive ESI with capillary voltage, 4000 V; end plate offset, 500 V; nebulizer pressure, 4 bar (N_2_); drying gas, 8 L/min (N_2_); and drying temperature, 200 °C.	[32]
EMPA	LC-UV: Kromasil 100 C18 column from Merck kGaA (Darmstadt, Germany) (4.6 × 250 mm, 5 µm) and isocratic elution with 10 mM ammonium acetate buffer in H_2_O:ACN (63:37, % *v*/*v*), 1 mL/min; UV 226 nm. UHPLC-MS: Eclipse plus C18 RRHD column from Agilent Technologies (2.1 × 50 mm, 1.8 µm) and a mobile phase comprising of H_2_O with 0.1% HCOOH and MeOH. LC-MS/MS: positive ESI mode with fragmentor voltage, 160 V; collision energy, 30 eV; capillary voltage, 3000 V; skimmer at 65 V; drying gas temperature (350 °C; 10 L/min); nebulizing gas (35 psi); temperature of sheath gas and flow (300 °C; 11 L/min); N_2_ as a drying gas.	[33]
IPRA	UPLC-UV: Hypersil Gold UPLC C18 column from Thermo Fisher Scientific (Waltham, MA, USA) (3 × 50 mm, 1.9 μm) and isocratic elution with 25 mM potassium phosphate of pH 3, adjusted using H_3_PO_4_ and ACN (50:50, *v*/*v*), 0.6 mL/min; UV 230 nm. Triple quadrupole-MS/MS: positive ion mode; capillary voltage 3 kV and the sampling cone voltage 20 V; the source temperature 120 °C, the desolvation temperature 400 °C, desolvation gas flow rate 600 L/h.	[34]

APCI—atmospheric pressure chemical ionization; TFA—trifluoroacetic acid.

## Data Availability

All data relevant to this study are included in the manuscript.
